# A Case of Alport Syndrome Associated with Recurrent Stanford Type B Aortic Dissections

**DOI:** 10.3400/avd.cr.22-00010

**Published:** 2022-06-25

**Authors:** Miki Takeda, Tadanori Minagawa, Wakiko Hiranuma, Takayuki Matsuoka, Takuya Shimizu, Shunsuke Kawamoto

**Affiliations:** 1 Department of Cardiovascular Surgery, Tohoku Medical and Pharmaceutical University, Sendai, Miyagi, Japan

**Keywords:** Alport syndrome, renal failure, Stanford type B aortic dissection

## Abstract

Alport syndrome is often characterized by renal dysfunction and hearing loss due to abnormalities in type IV collagen production. In this study, we report a rare case of recurrent aortic dissections that developed in a young patient with Alport syndrome over a short period. We discuss the associations between Alport syndrome and aortic dissection with a literature review and emphasize the need for regular follow-up of patients with Alport syndrome for early detection of aortic disease.

## Introduction

Alport syndrome has been described as a genetic disorder caused by pathogenic mutations in the collagen type IV alpha 3 (COL4A3), collagen type IV alpha 4 (COL4A4), or collagen type IV alpha 5 (COL4A5) genes encoding type IV collagen α3, α4, or α5 chains, respectively. It causes a systemic disorder of type IV collagen in the basement membranes and then manifests as glomerulonephritis, which, in turn, leads to renal dysfunction even at a young age.^1)^ Although numerous cases of renal dysfunction and hearing impairment with Alport syndrome have been reported, reports on arterial pathologies have remained few. Thus, in this study, we report a case of a patient with Alport syndrome who developed aortic dissection twice over a short duration.

## Case Report

**Figure 1:**
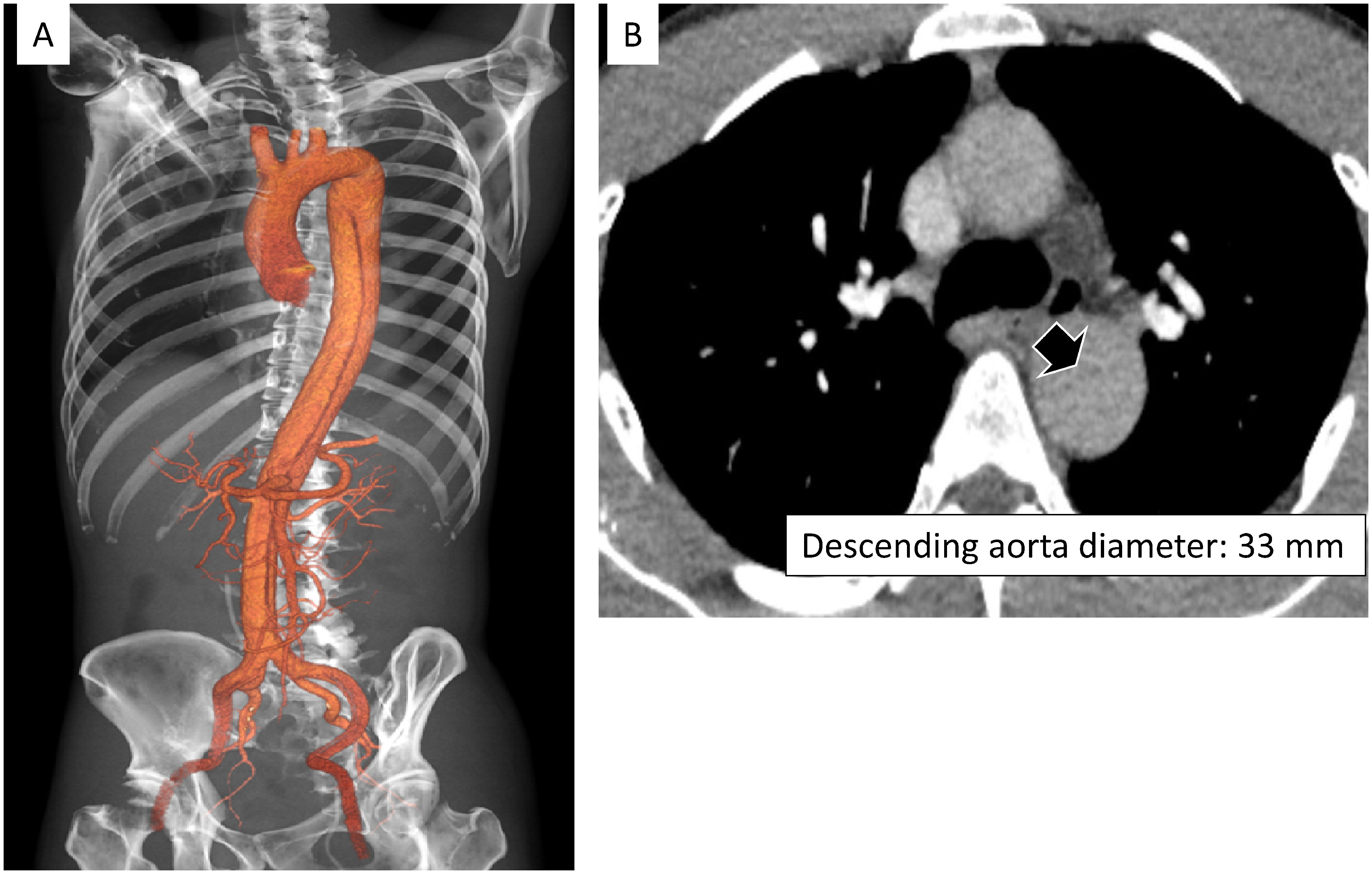
Fig. 1 (**A**) Three-dimensional computed tomography (CT) angiography on the day of onset. (**B**) CT on the day of onset of aortic dissection. Maximum axial diameter of the descending aorta, 33 mm. Arrow: flap.

A 41-year-old man experienced severe acute back pain and was transferred to our hospital. He was diagnosed with Alport syndrome at 13 years of age via renal biopsy, which showed the deposition of immunoglobulin A (IgA) and IgM in the mesangium and loop wall by immunofluorescence. Type IV collagen α2 and α5 showed decreased staining, with α5 staining showing a loss of the basement membrane of the Bowman’s capsule. The glomerular basement membrane also showed segmental loss of staining. Based on these results, X-linked Alport syndrome (XLAS) was diagnosed. His comorbidities included hypertension, rheumatoid arthritis, atopic dermatitis, and chronic renal failure. His blood pressure was 187/118 mmHg on presentation to the emergency room. Contrast-enhanced computed tomography (CT) revealed acute Stanford type B aortic dissection from the origin of the left subclavian artery to the bilateral external iliac arteries (**Fig. 1A**). The bilateral renal arteries originated from the true lumen, whereas the inferior and superior mesenteric arteries originated from the false lumen without malperfusion. The aortic diameters at the distal arch, descending aorta, and abdominal aorta were 33, 33, and 23 mm, respectively. The aortic root was observed to be not enlarged (**Fig. 1B**). The patient was admitted to the intensive care unit and administered antihypertensive and analgesic therapy. His renal function deteriorated temporarily, but hemodialysis was not required. During hospitalization, his systolic blood pressure was approximately 100–120 mmHg at rest; after rehabilitation, it stabilized to approximately 120 mmHg. Antihypertensive drug regimen included azilsartan (20 mg) and trichlormethiazide (1 mg). The aortic diameter did not change remarkably at discharge on day 40.

**Figure 2:**
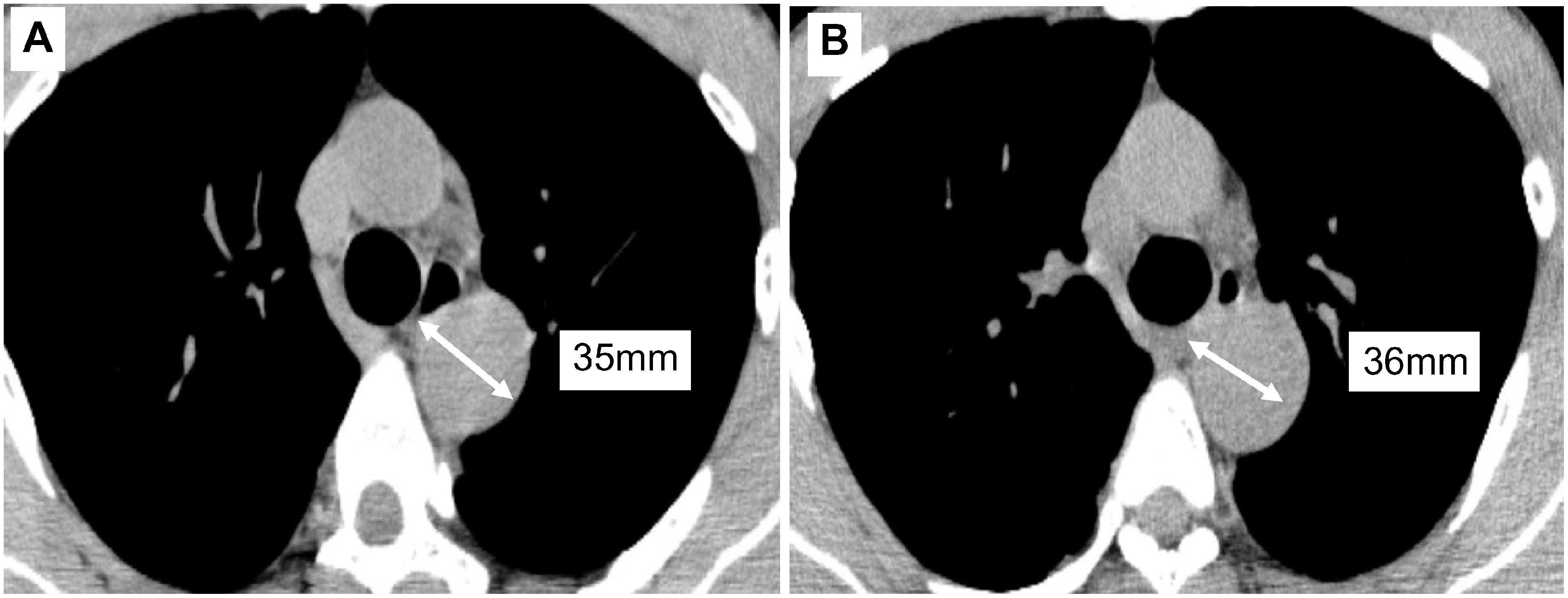
Fig. 2 (**A**) Computed tomography (CT) at 3 months after discharge. Maximum axial diameter of the descending aorta, 35 mm. (**B**) CT performed at 9 months after discharge showing the maximum axial diameter of the descending aorta as 36 mm.

During outpatient follow-up at 3 and 6 months after onset, his blood pressure was well controlled; however, periodic CT scans showed slight dilatation of the descending aorta with an aortic diameter of 35 mm at 3 months after the first onset (**Fig. 2A**), which subsequently increased to 36 mm 3 months later (**Fig. 2B**).

**Figure 3:**
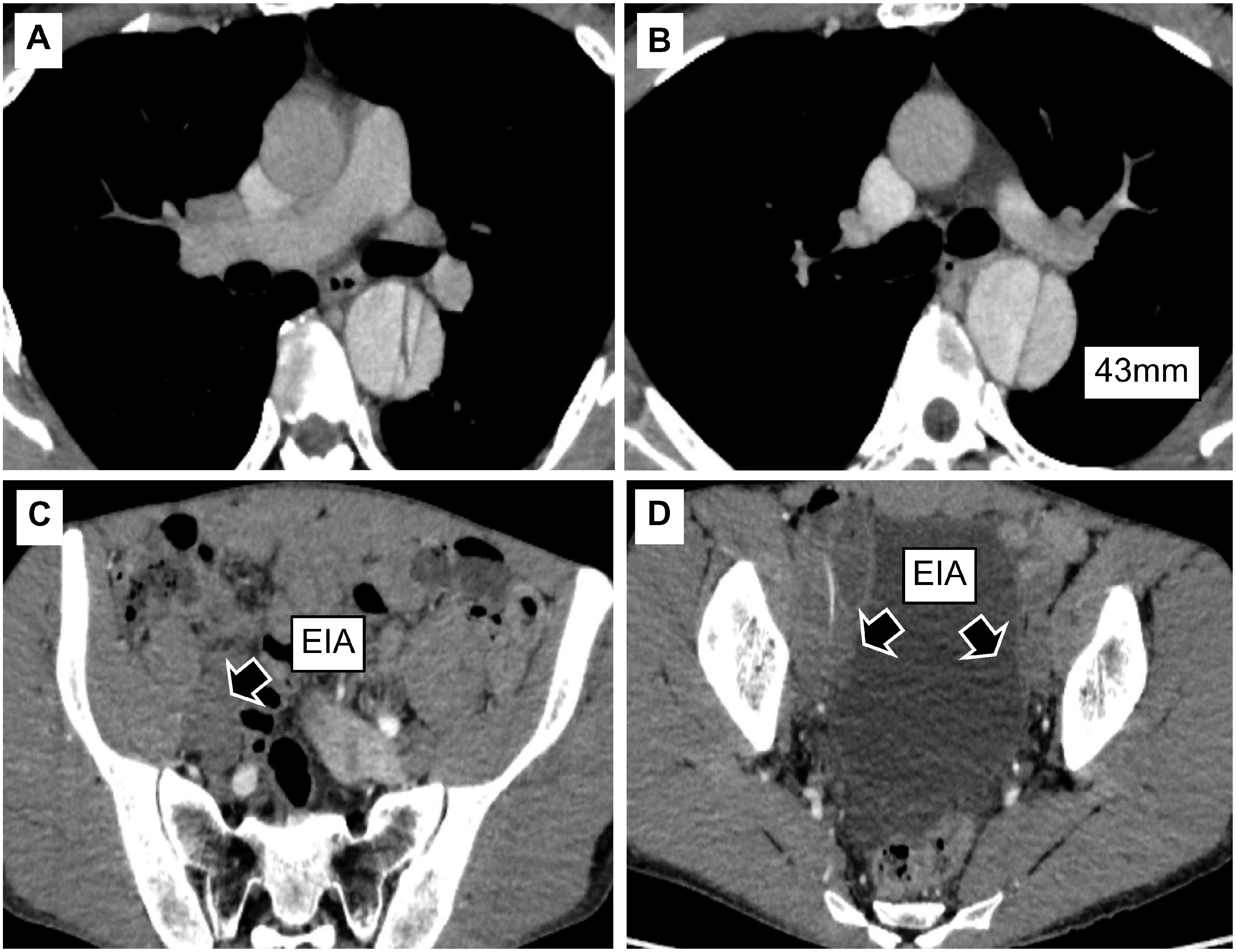
Fig. 3 (**A**) Computed tomography showing the second Stanford type B acute aortic dissection after 16 months since the onset of the first aortic dissection. (**B**) The maximum axial diameter of the descending aorta was 43 mm. (**C**) The blood flow in the external iliac artery (EIA) was disrupted. Arrow: The left EIA. (**D**) Disrupted blood flow of the left and right EIAs.

Sixteen months after the onset of the first aortic dissection, the patient presented with back and neck pain and severe left lower limb numbness. His bilateral femoral and popliteal arteries were not palpable, and severe cyanosis and impaired muscular movement were observed in the left lower limb, although the touch sensation was preserved. His blood pressure on presentation to the emergency room was 212/106 mmHg. Contrast-enhanced CT revealed a second Stanford type B aortic dissection. The true lumen of the descending aorta was stenosed with complex dissection of the false lumen (**Fig. 3A**), and the maximum diameter of the distal arch was 43 mm (**Fig. 3B**). Although blood flow in the external iliac artery was disrupted (**Figs. 3C** and **3D**) and bilateral femoral artery perfusion was noted due to collateral circulation, no other malperfusion was observed. Thoracic endovascular aortic repair (TEVAR) is not recommended in patients with connective tissue disorders. Since the patient had a complicated aortic dissection with three lumens in some parts, TEVAR was expected to be complicated. We thus considered axillobifemoral bypass as the best option to save the patient’s life in the shortest possible time. To salvage his lower limbs, an emergency right axillary-bilateral femoral artery bypass surgery was performed. Perioperative examination revealed abnormally thin arterial walls, which indicate the vulnerability of the vessels. As there was no unwanted tissue, histopathological examination was not performed in this surgery. The surgery was performed without any major complications. His postoperative maximum creatine phosphokinase level was 10,892 U/L, and his serum potassium level reached 6.7 mEq/L; however, sufficient volume load therapy contributed to urine output maintenance and a decrease in serum potassium level; renal replacement therapy was not required for recovery. The postoperative course was deemed uneventful, and his lower limb function improved. However, his blood pressure was noted to be higher than that during his previous hospitalization. An antihypertensive regimen comprising amlodipine (2.5 mg), nifedipine (80 mg), valsartan (80 mg), and doxazosin mesylate (2 mg) was administered. His CT scan showed no further dilatation of the dissecting aorta, and he was discharged on day 29. Even after 2 years, he has been closely observed for his aorta with periodic CT examinations, and aortic surgery will be considered if further dilatation of the aorta is observed.

## Discussion

This study describes a case of XLAS in a patient with poorly controlled blood pressure and repeated aortic dissection (twice within 16 months). Although the blood pressure of the patient was stable during the first hospitalization, it was difficult to control during outpatient visits and daily life.

XLAS is characterized by mutations in COL4A5, the gene encoding the α5(IV) chain; in most men with XLAS, the basement membrane lacks the expression of α3α4α5 and α5α5α6 trimers.^2)^ The absence of α3α4α5 trimers in the glomerular basement membrane induces structural and functional abnormalities of the glomerulus, which could ultimately lead to renal fibrosis. Therefore, in Alport syndrome, protective strategies for renal function are determined to be clinically important, and therapeutic interventions are often performed for renal hypofunction and hearing loss, but screening for aortic disease is rarely performed. Kashtan et al. described the effect of targeted mutations in COL4A5 on the expression of α5(IV) chains in the mouse aorta, suggesting that the presence of a type IV collagen network composed of α5(IV) and α6(IV) chains plays a role in maintaining the integrity of aortic blood vessels.^3)^ In other words, type IV collagen is involved in the mechanism of aortic dissection, and the lack of type IV collagen in Alport syndrome may cause vascular complications earlier than in people without Alport syndrome. Until now, there have been no reports of arterial wall samples from humans with Alport syndrome, so the actual pathology of the arterial wall is yet to be known. However, since Alport syndrome is caused by a genetic problem, it is expected that the basement membrane of the human aorta is also deficient in collagen IV, and if there is an opportunity in the future, we would like to collect arterial wall tissue for investigation.

In the presented case, although arterial enlargement after the first aortic dissection was mild, a second aortic dissection occurred. Patients with Alport syndrome may develop aortic dissection, regardless of aortic diameter, due to vascular vulnerability. Therefore, they require stricter blood pressure control than other patients. CT and echocardiography should also be performed in patients with Alport syndrome, Marfan syndrome, and other collagen diseases. Lyons et al. reported aortic aneurysm rupture in patients with Alport syndrome and recommended that CT scans be performed even in younger patients.^4)^ Furthermore, Vogt et al. reported a poor prognosis for aortic dissection in young patients with chronic hypertension; all their patients had hypertension associated with chronic renal failure.^5)^ This current report also shows that renal hypertension is difficult to control. Patients with Alport syndrome are thought to be more prone to aortic disease because of the combination of renal hypertension and vascular fragility.

Aggressive use of ACE inhibitors (ACEIs) and angiotensin receptor blockers (ARBs) has been recommended for blood pressure control in patients with Alport syndrome.^6)^ This is because the EARLY PRO-TECT ALPORT trial showed that both ACEIs and ARBs are effective in decreasing urinary protein levels, which then prevents renal function decline.^7)^ A large retrospective study by Gross et al. reported that ACEIs slow down the progression to end-stage renal failure and improve life expectancy in patients with Alport syndrome.^7)^ Moreover, ACEIs and ARBs prevent renal hypertension and cardiovascular complications by reducing renal hypofunction and preventing blood pressure elevation, respectively. Although there is no comparative study on a single administration of either ACEI or ARB alone versus combined administration of both ACEI and ARB, theoretically, combined therapy may provide stronger renal protection in Alport syndrome.

## Conclusion

Patients with Alport syndrome may be more susceptible to aortic disease due to the vulnerability of blood vessels associated with type IV collagen abnormalities and difficulty in controlling blood pressure due to renal hypertension. Early diagnosis of Alport syndrome and renal protection can prevent this vicious cycle. Therefore, preventing the worsening of renal function using both ACEIs and ARBs remains a priority. This will help prevent renal hypertension. Even at an early age, patients should be followed up for aortic disease via CT and echocardiography. Additionally, the possibility of Alport syndrome should be considered in young patients with aortic dissection.
